# Glans Diameter and Meatus Localization Are the Sole Predictors of Primary Distal Hypospadias Surgery Complications: A Multivariate Analysis of Single Surgeon Series

**DOI:** 10.7759/cureus.30306

**Published:** 2022-10-14

**Authors:** Fatih Akova, Emrah Aydın, Emre Salabas, Zeynep Bilgili

**Affiliations:** 1 Department of Pediatric Surgery, Biruni University, Istanbul, TUR; 2 Department of Pediatric Surgery, Namık Kemal University School of Medicine, Tekirdağ, TUR; 3 Department of Urology, University of Health Sciences, Sisli Hamidiye Etfal Training and Research Hospital, Istanbul, TUR

**Keywords:** hypospadias surgery, tipu, tubularized incised plate urethroplasty, distal, predictors, complications, surgery, hypospadias

## Abstract

Introduction: Tubularized incised plate urethroplasty (TIPU) surgery is among the most successful techniques for distal hypospadias. Our objective was the investigation of complication rates and their predictors.

Methods: Between 2010 and 2021, 150 patients with distal hypospadias were operated on consecutively by a single surgeon using the TIPU technique. The primary outcome was the complication rates including fistula, meatal stenosis, and glans dehiscence. Secondary outcomes were predictor factors of complications.

Results: The average glans diameter was 13.9 ± 0.10 mm and 57.0% of the patients had a glans diameter greater than 14 mm. Single-layer and double-layer urethroplasty were used in 55.3% (n = 83) and 44.7% (n = 67) of patients, respectively. Overall complication rate was 23.3% (n = 35), which included fistula (3.3%, n = 5), glans dehiscence (12.7%, n = 19), and meatal stenosis (8.6%, n = 13). Glandular meatus localization (OR = 58.8, p = 0.001) and smaller glans diameter (OR = 0.39, p = 0.001) were significant predictors in the multivariate analysis of overall complications. For fistula complications, only short operation time (OR = 0.83, p = 0.03) was found as a significant predictor. Glans width (<14 mm) was the only significant predictor of both glans dehiscence (OR = 3.4, p = 0.03) and stenosis (OR = 5.67, p = 0.013) complication.

Conclusion: TIPU technique for distal hypospadias has notable success and acceptable complication rates. Dartos augmented single-layer urethral closure seems adequate for complication prevention. Preoperative assessment of the glans width and meatus site is advised to predict complication rates.

## Introduction

Hypospadias, with an incidence that varies from 0.3% to 0.8% in live male births, is one of the most frequent congenital urogenital abnormalities [1,2]. Hypospadias surgery is among the most challenging conditions in pediatric surgery practice. Although there have been many improvements in instrumentation, suture materials, anatomical knowledge, and surgical details, a surgical golden standard equally applicable to all levels of hypospadias is still lacking. The existence of more than 300 different surgical procedures is a testament to this surgical challenge [1]. A variety of differentiating elements such as the size of the penis and urethral plate, level of division of the corpus spongiosum, presence of curvature, and position of the scrotum add to the complexity of these cases [1,2].

More than 50% of hypospadias patients have their meatus located distally and tubularized incised plate urethroplasty (TIPU) repair is the most preferred surgical technique for those [2]. This technique also proved its mettle in proximal and recurrent hypospadias cases [3]. TIPU repair, among many others, has obtained widespread acceptance for having low complication rates and better cosmetic and functional outcomes for distal hypospadias. Fistula, glans dehiscence, and meatal stenosis are the most common complications of this surgery [4]. Identifying the potential risk factors for these complications is required to improve our surgical success and inform our patients with precise data. We aimed to analyze the possible patient and technique-based predictors, for both overall complications and their subgroups.

## Materials and methods

After Institutional Review Board's approval was obtained, a retrospective chart review was performed on all hypospadias patients from 2010 to 2021. Data were collected on patient demographics, surgical procedures, and outcomes. The inclusion criteria were patients who had TIPU for distal hypospadias with a penile chordee of less than 20 degrees. The exclusion criteria were patients with proximal hypospadias surgeries and penile chordee of more than 20 degrees.

The primary outcome was the complication rates including fistula, meatal stenosis, and glans dehiscence. The fistula was defined as urinary leakage, and meatal stenosis was defined as difficulty in calibration with the feeding diameter used in the operation (either 6 Fr or 8 Fr) on the 21st postoperative day, and because of the final stage of the wound healing, remodeling begins approximately three weeks after surgery [5]. Glans dehiscence complication (GDC) included both complete and partial separations. Complete glans dehiscence was defined similarly to Dr. Snodgrass’s article, i.e., a complete separation of glans wings resulting in coronal or sub-coronal meatus and requiring a reoperation [4]. Partial glans separation was defined as the opening of the neourethral ventral lip up to 2 mm. Secondary outcome measures were surgical complication predictor factors such as age, follow-up time, meatus localization, the existence of chordee, glans diameter, catheter diameter, urethral closure (single or double layer), and operation time.

**Figure 1:**
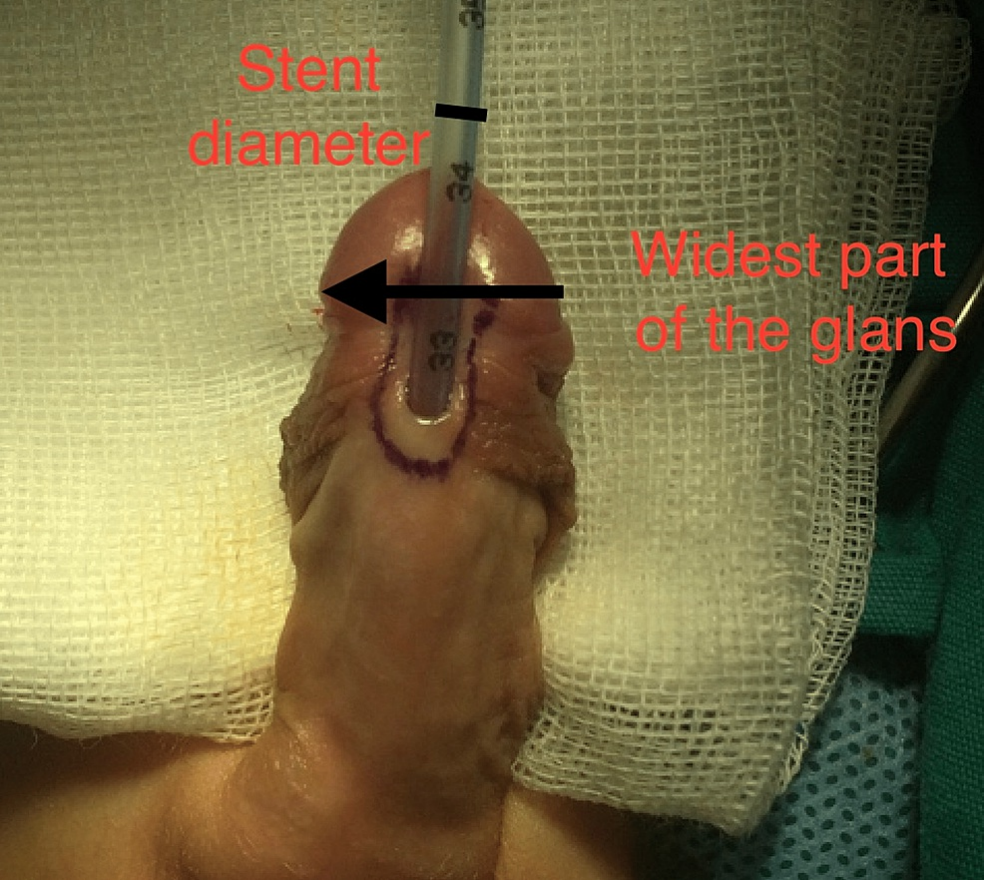
Ratio of the widest part of the glans to the stent diameter Glans diameter = (widest part of the glans/stent diameter) x stent (6 or 8 Fr) diameter (mm). 1 Fr = 1/3 mm.

Glans diameter was measured from the intraoperative patient photographs by proportioning of feeding catheter width to the glans width. Photographs of the penis and glans were taken for all patients at the beginning of the operation and without an erection. These photographs were taken digitally from the ventral side of the penis using the same machine. The lens of the camera was always parallel to the ventral side of the penis from the same distance. The pictures were evaluated in the digital environment and the ratio of the widest part of the glans to the stent diameter was found. Glans diameter was obtained by multiplying this ratio with the stent diameter, a constant value (Figure 1). Small glans diameter was defined as smaller than 14 mm, similar to studies by Snodgrass et al. [6].

Surgical technique

The surgery was performed over a urethral catheterization as a stent for the neo-urethra, which was either 6 Fr or 8 Fr depending on the patient’s age. A U-shaped incision that encircles the urethral meatus followed the catheterization while preserving the urethral plate. Complete penile degloving was done, which was followed by chordee testing and correction of the chordee if needed. Intraoperative artificial erection and protractor-assisted curvature measurements were performed on all patients with suspected penile curvature.

A tourniquet was applied for 20 minutes at most on the base of the penis to minimize primary hemorrhage during urethroplasty. A vertical incision was done on the urethral plate. The urethral plate incision depth was performed depending on the width of the urethra (reaching corpora if necessary) and was extended to the proximal to the native urethral meatus. Glans wing incisions were also extended to the corpus cavernosum. Tubularization sutures of the urethra were placed in an interrupted fashion from proximal to distal with 7-0 polydioxanone (PDS) suture in either one or two layers, followed by a dorsal dartos flap that covered the neo-urethra. The ventral lip (1 mm) of neo-urethra was intentionally left open to reduce meatal stenosis. The dorsal dartos flap was fixated beneath the glans wings with a 6-0 PDS suture, followed by glans wings closure with a 6-0 PDS suture. Two layers of sutures, 3-4 submucosal inverting and 4-5 extra-mucosal sutures, were placed to approximate the glans wings. Circumcision was performed as a routine part of the procedure in all patients. The urethral catheters were removed on postoperative day seven. On postoperative day 21, urethral meatus calibration was controlled via a urethral catheter to exclude stenosis. Calibration was done by applying local lidocaine gel. Meatal stenosis was suspected if difficulty occurred during calibration. In the case of suspected meatal stenosis, parents performed daily urethral dilatation for a month and were followed up at the clinic for control after two months. All of the patients previously suspected of meatal stenosis passed the calibration test in their second clinic visit at three months. Patients were invited to routine follow-ups for the late complications of hypospadias surgery up to five years in six months intervals.

Statistical analysis

Statistical analysis was performed with IBM SPSS Statistics 24.0.0 (IBM Corp., Armonk, NY). The attributes of the study sample were summarized by descriptive statistics, with dichotomous or ordinal data presented as percentages, and continuous data as means with standard deviations or median with interquartile range (IQR) for non-normal distribution. Shapiro-Wilk test, skewness, and kurtosis values were applied to demonstrate normal distribution. Student’s t-test and Pearson correlation were used for parametric statistics, and Mann-Whitney U and Spearman correlation were used for non-parametric statistics. Levine's tests of the Student's t-tests were all non-significant. We utilized binary multivariate logistic regression to estimate surgery complication odds. A backward multivariate regression model was preferred. The model was generated from risk factors with a significant odds ratio or p-values < 025, calculated in univariate regression analysis. We considered statistical associations significant if the p-value was < 0.05 and the confidence intervals were 95%.

## Results

Between 2010 and 2021, 150 patients with distal hypospadias were operated on consecutively by a single surgeon using the TIPU technique. None of the patients were lost on follow-up.

The patients' median age and follow-up time were 19 months (IQR = 12-48) and 74 months (IQR = 25-98), respectively. The mean operation time was recorded as 95.4 + 0.90 minutes. The average glans diameter was 13.9 + 0.10 mm and 57.0% of the patients had a glans diameter greater than 14 mm. Chordee less than 20 degrees was detected in 34% (n = 51) of patients during the operation. Meatus was in the glandular-coronal area in 48.7% (n = 73) and the more proximal (distal-midshaft) in 51.3% (n = 77) of patients. Single-layer urethroplasty plus dartos closure was employed for over 55.3% (n = 83) of patients while an 8 Fr catheter was applied in approximately one-third of patients (Table 1). Overall complication rate was 23.3% (n = 35), which included fistula (3.3%, n = 5), glans dehiscence (12.7%, n = 19), and meatal stenosis (8.6%, n = 13) (Table 2). Glans dehiscence included three complete separations (2%) and 16 (10.7%) partial separations. Two of the meatal stenosed patients also developed fistula complications. These two fistulas closed after periodic urethral dilatation of stenosis. The remaining three fistulas and three complete glans dehiscence had reoperations (n = 6, 4%). Two groups were created comparing patients with and without complications. These groups were retrospectively investigated for patient and operation parameters.

**Table 1:** Patient and surgery parameter statistics IQR = interquartile range for median values; Fr = French.

Patient and surgery parameter statistics		
Number of patients	150	Skewness/kurtosis
Median age/IQR (months)	19.0 (12-48)	1.49 + 0.20/1.77 + 0.39
Median follow-up/IQR (months)	74 (25-98)	-0.21 + 0.20/-1.31 + 0.39
Mean glans diameter (mm)	13.9 + 0.10	-0.21 + 0.20/0.76 + 0.39
Mean operation time (minutes)	95.4 + 0.90	0.36 + 0.20/-0.13 + 0.39
	n (%)	
Glans diameter: <14 mm, >14 mm	65 (43.0%), 86 (57.0%)	
Chordee: absent, present	99 (66.0%), 51 (34.0%)	
Meatus localization: glandular-coronal, distal-midshaft	73 (48.7 %), 77 (51.3%)	
Catheter: 6 Fr, 8 Fr	98 (65.3%), 52 (34.7%)	
Single layer, double layer	83 (55.3%), 67 (44.7%)	

**Table 2:** Comparison of patients with and without complications (overall and subgroups) P-values are written in bold style if p ≤ 0.05. IQR = interquartile range for median values; Fr = French.

	Overall complication (-)	Overall complication (+)	p	Fistula (-)	Fistula (+)	p	Glans dehiscence (-)	Glans dehiscence (+)	p	Stenosis (-)	Stenosis (+)	p
N (%)	115 (76.7%)	35 (23.3%)	0.001	145 (96.7%)	5 (3.3%)	0.001	131 (87.3%)	19 (12.7%)	0.001	137 (91.4)	13 (8.6%)	0.001
Median age/IQR (months)	23 (10-60)	12 (10-34)	0.017	20 (12-48)	16 (9-36)	0.34	23 (12-48)	12 (10-24)	0.024	18 (12-48)	24 (10-48)	0.58
Follow-up/IQR (months)	77 (33-99)	50 (12-94)	0.012	75 (25-98)	50 (9-76)	0.18	75 (28-98)	45 (16-82)	0.075	75 (26-98)	50 (9-95)	0.15
Meatus localization			0.001						0.001			0.001
Glanular-coronal	75 (97.4%)	2 (2.6%)		75 (97.4%)	2 (2.6%)	0.67	77 (100%)	0 (0%)		77 (100%)	0 (0%)	
Distal-midshaft	40 (54.8%)	33 (45.2%)		70 (95.9%)	3 (4.1%)		54 (74%)	19 26%)		60 (82.2%)	13 (17.8%)	
Chordee -	78 (78.8%)	21 (21.2%)	0.39	95 (96.0%)	4 (4.0%)	0.66	87 (87.9%)	12 (12.1%)	0.78	93 (93.9%)	6 (6.1%)	0.11
Chordee +	37 (72.5%)	14 (27.5%)		50 (98.0%)	1 (2.0%)		44 (86.3%)	7 (13.7%)		44 (86.3%)	7 (13.7%)	
Glans diameter			0.001						0.015			
<14 mm	39 (60.9%)	25 (39.1%)		60 (93.8%)	4 (6.3%)	0.164	51 (79.7%)	13 (20.3%)		54 (84.4%)	10 (15.6%)	0.01
>14 mm	76 (88.4%)	10 (11.6%)		85 (98.8%)	1 (1.2%)		80 (93%)	6 (7%)		83 (96.5%)	3 (3.5%)	
Glans diameter (mm)	14.2 + 1.2	13.4 + 1.1	0.005	14.0 + 1.2	12.7 + 1.0	0.014	14.1 + 1.10	13.6 + 1.2	0.085	14.1 + 1.2	13.4 + 0.9	0.05
Catheter												
6 Fr	70 (71.4%)	28 (28.6%)	0.037	93 (94.9%)	5 (5.1%)	0.164	82 (83.7%)	16 (16.3%)	0.06	89 (90.8%)	9 (9.2%)	1
8 Fr	45 (86.5%)	7 (13.5%)		52 (100%)	0 (0%)		49 (94.2%)	3 (5.8%)		48 (92.3%)	4 (7.7%)	
Urethral closure												
Single layer	68 (81.9%)	15 (18.1%)	0.09	79 (95.2%)	4 (4.8%)	0.38	76 (91.6%)	7 (8.4%)	0.08	77 (92.8%)	6 (7.2%)	0.5
Double layer	47 (70.1%)	20 (29.9%)		66 (98.5%)	1 (1.5%)		55 (82.1%)	12 (17.9%)		60 (89.6%)	7 (10.4%)	
Operation time (min)	94.7 + 10.8	97.7 + 10.9	0.06	95.8 + 10.8	84.0 + 8.9	0.02	92.9 + 10.7	99.4 + 11.0	0.06	95.1 + 10.9	98.4 + 9.4	0.11

Overall complications

Patients who developed a complication (n = 35, 23.3%) were compared with patients who did not have any complications (n = 115, 76.7%). As indicated in Table 2, median age (12, IQR = 10-34 vs. 23, IQR = 10-60 months, p = 0.017) and follow-up time (50, IQR = 12-94 vs. 77, IQR = 33-99 months, p = 0.012) were statistically lower in the complication group. Glans diameter average and percentage of patients with glans diameter > 14 mm was significantly lower in the complication group: 13.4 + 1.1 mm vs. 14.2 + 1.2 mm (p = 0.005) and 11.6% vs. 39.1% (p = 0.001), respectively. Almost all complications developed in patients with glandular-coronal meatus rather than mid-distal penile shaft-located meatus (45.2% vs. 2.6%, p = 0.001). Higher operation time had a tendency of greater complication rate association (97.7 + 10.9 vs. 94.7 + 10.8 minutes, p = 0.06). Smaller catheter usage (6 Fr vs. 8 Fr) was significantly associated with a higher complication rate (28.6% vs. 13.5%, p = 0.04) (Table 2).

Fistula complication

In this subgroup analysis, two groups included patients who developed a fistula (n = 5, 3.3%) and those who did not (n = 145, 96.7%). The average glans diameters of patients with fistula were significantly smaller than the other group (12.7 + 1.0 vs. 14.0 + 1.2 mm, p = 0.014). Only other significant factor was shorter operation time for fistula group (84.0 + 8.9 minutes vs. 95.8 + 10.8 minutes, p = 0.02) (Table 3). None of the remaining parameters showed a significant discrepancy between the two groups (Table 2).

**Table 3:** Binary logistic regression analysis for complications (overall and subgroups) P-values are written in bold style if p ≤ 0.05.

	Overall complications					Fistula complications					Glans dehiscence							Meatus stenosis				
	Univariate analysis		Multivariate analysis		Univariate analysis			Multivariate analysis		Univariate analysis				Multivariate analysis			Univariate analysis			Multivariate analysis		
	OR (95% CI)	p	OR (95% CI)	p	OR (95% CI)		p	OR (95% CI)	p	OR (95% CI)		p	OR (95% CI)		p	OR (95% CI)			p	OR (95% CI)	p	
Age	0.98 (0.96-1.00)	0.032	-	-	0.98 (0.93-1.03)		0.39	-	-	0.97 (0.94-1.00)		0.05	-		-	0.99 (0.97-1.02)			0.62	-	-	
Follow up	0.98 (0.97-0.99)	0.011	-	-	0.99 (0.96-1.00)		0.21	-	-	0.99 (0.98-1.00)		0.065			-	0.98 (0.98-1.00)			0.16	-	-	
Meatus local.	30.93 (7.05-135.6)	0.001	58.8 (10.66-324.20)	0.001	0.62 (0.10-3.83)		0.61	-	-	0.000		0.96	-		-	0.000			0.99	-	-	
Coronal-glanular																						
Chordee +	1.40 (0.64-3.01)	0.39	-	-	0.48 (0.05-4.36)		0.51	-	-	1.15 (0.42-3.13)		0.78	-		-	2.46 (0.78-7.77)			0.12	-	-	
Glans diameter	4.87 (2.12-11.16)	0.001	-	-	5.66 (0.61-51.97)		0.13	-	-	3.39 (1.21-9.21)		0.02	3.4 (1.12-10.34)		0.03	5.13 (1.35-19.47)			0.016	5.67 (1.45-21.72)	0.013	
<14 mm																						
Glans (mm)	0.56 (0.49-0.89)	0.001	0.39 (0.25-0.65)	0.001	0.42 (0.20-0.87)		0.02	-	-	0.70 (0.47-1.05)		0.09	-		-	0.63 (0.39-1.01)			0.06	-	-	
Catheter 6 Fr	2.57 (1.04-6.39)	0.04	-	-	0.000		0.99	-	-	0.31 (0.09-1.13)		0.08	-		-	0.83 (0.24-2.81)			0.76	-	-	
Urethral closure	1.93 (0.89-4.26)	0.09	-	-	0.29 (0.03-2.74)		0.29	-	-	2.37 (0.87-6.40)		0.09	-		-	1.50 (0.48-4.68)			0.48	-	-	
Double layer																						
Operation time (min)	1.03 (0.99-1.06)	0.15	-	-	0.87 (0.78-0.98)		0.02	0.83 (0.70-0.98)	0.03	1.04 (0.99-1.08)		0.09	-		-	1.03 (0.97-1.08)			0.29	-	-	

Glans dehiscence complication

GDC occurred in 12.7% (n = 19) of 150 hypospadias patients. Patients with GDC were significantly younger than their counterparts (12, IQR = 10-24 vs. 23, IQR = 12-48 months, 0.024) and had a lower follow-up time (45, IQR = 16-82 vs. 75, IQR = 28-98 months, 0.075). All the patients with GDC had their meatus at glandular-coronal level (26% vs. 0%, p = 0.001). The majority of the patients in the GDC group had a glans diameter smaller than 14 mm (20.3% vs. 7%, p = 0.015). The average glans diameter was also smaller in the complication group, but it did not reach statistical significance (13.6 + 1.2 vs. 14.1 + 1.1, 0.085). Six Fr catheters (16.3% vs. 5.8%, 0.06) and double layer urethral closure (17.9% vs. 8.4%, p = 0.08) were notably predominant in the GDC group but neither showed statistical significance. Patients with GDC had a non-significant but higher operation time than their counterparts (99.4 + 11.0 minutes vs. 92.9 + 10.7 minutes, p = 0.06) (Table 2).

Stenosis complication

Thirteen out of 150 patients developed postoperative stenosis (8.6%) and all of their meatuses were in glandular-coronal locations (17.8% vs. 82.2%, p = 0.001). Both average glans diameter (13.4 + 0.9 mm vs. 14.1 + 1.2 mm, p = 0.05) and frequency of patients with glans diameter threshold of 14 mm (15.6% vs. 3.5%, 0.01) showed statistical disparity between stenosis complication and non-stenosis complication groups. Stenosis complications arose in patients with smaller glans, which were generally smaller than 14 mm. None of the other parameters showed a significant variation between the two groups (Table 2).

Predictors for overall complications

In univariate regression analysis for overall complications, follow-up time (OR = 0.98, 95% CI = 0.97-0.99, p = 0.011), glandular localization (OR = 30.93, 95% CI = 7.05-135.6, p = 0.001), glans diameter < 14 mm (OR = 4.87, 95% CI = 2.12-11.16, p = 0.001), average glans diameter (OR = 0.56, 95% CI = 0.49-0.89, p = 0.001), and 6 Fr catheter (OR = 2.57, 95% CI = 1.04-6.39, p = 0.04) were all significant factors. However, in the binary multivariate regression analysis model, formed from significant univariate parameters, only glandular meatus localization (OR = 58.8, 95% CI = 10.66-324.20, p = 0.001) and average glans diameter (OR = 0.39, 95% CI = 0.25-0.65, p = 0.001) were significant predictors (Tables 3, 4). In other words, estimated odds for a complication decreased by 61% for every 1 mm increase in glans diameter.

**Table 4:** Summary table of regression analysis of complications UV: univariate analysis; MV: multivariate analysis; OR: odds ratio; CI: confidence interval; Fr: French.

	Overall complication		Fistula		Glans dehiscence		Stenosis	
OR/p	UV	MV	UV	MV	UV	MV	UV	MV
Age		-	-	-		-	-	-
	Follow up				0.98				-	-	-	-	-	-	-
0.01															
Meatus local.	30.93	58.8	-	-	-	-	-	-
Glanular	0.001	0.001						
Chordee +	-	-	-	-	-	-	-	-
Glans diameter	4.87	-	-	-	3.39	3.4	5.13	5.67
<14 mm	0.001				0.02	0.03	0.02	0.01
Glans diameter (mm)	0.56	0.39	0.42	-	-	-	-	-
	0.01	0.001	0.02					
Catheter 6 Fr	2.57	-	-	-	-	-	-	-
	0.04							
Urethral closure	-	-	-	-	-	-	-	-
Double layer								
Operation time (min)	-	-	0.87	0.83	-	-	-	-
			0.02	0.03				

Predictors for fistula complications

In univariate regression analysis for fistula complications, only average glans diameter (OR = 0.42, 95% CI = 0.20-0.87, p = 0.02) and operation time (OR = 0.87, 95% CI = 0.78-0.98, p = 0.02) were significant predictors. However, in the binary multivariate regression analysis model, only operation time was found as the predictor for fistula complications (OR = 0.83, 95% CI = 0.70-0.98, p = 0.03) (Tables 3, 4). Estimated odds for a fistula complication decreased by 13% for every one-minute increase in operation time.

Predictors for GDC

In univariate regression analysis for GDC, glans diameter < 14 mm (OR = 3.39, 95% CI = 1.21-9.21, p = 0.02) was found as a significant predictor. However, in the binary multivariate regression analysis model, only glans diameter < 14 mm was found as a significant predictor (OR = 3.4, 95% CI = 1.12-10.34, p = 0.03) (Tables 3, 4). Explicitly estimated odds for a GDC increased by 340% for patients with a glans diameter < 14 mm.

Predictors for stenosis complications

In univariate regression analysis for stenosis complication predictors, only glans diameter < 14 mm (OR = 5.13, 95% CI = 1.35-19.47, p = 0.016) was a significant predictor. In the binary multivariate regression analysis model, glans diameter < 14 mm (OR = 5.67, 95% CI = 1.45-21.72, p = 0.013) was still the only significant predictor of stenosis complication (Tables 3, 4). There was a 560% estimated increase in the odds of patients with a glans diameter smaller than 14 mm for stenosis complication.

## Discussion

An ideal hypospadias surgery should have excellent cosmetic and functional results with minimal complications [7]. However, there is a broad range of complications in the literature including fistula, urethral and meatal stenosis, glans/wound dehiscence, and curvature persistence [8]. While the overall complication rate was reported up to 43% for distal cases, a pooled estimate of overall complication was reported as 8% (95% CI: 6.3-9.8%) in a recent meta-analysis investigating non-proximal cases [7,8]. Our overall complication rate of 23.3% (Table 2) is relatively high in comparison to the literature but only 4% of the patients required a reoperation (for fistula and complete glans dehiscence). This high overall complication rate is contributed to high partial glans dehiscence and meatal stenosis rates, which will be explained in their respective paragraphs. Younger children (12 vs. 23 months) with smaller glans diameters (13.4 mm vs. 14.2 mm) and urethral catheters (6Fr vs. 8Fr), and coronally located meatus had a significantly higher rate of complication in our study. In our multivariate analysis of overall complication predictors, glandular-coronal meatus and glans diameter (mm) were the only significant independent predictors. In another TIPU series by Snodgrass et al., predictors of urethral complications were small glans size (<14 mm), reoperations, and proximal/mid meatus [6]. The complications were similarly higher in patients with small glans (<14 mm) than patients with glans > 14 mm in both studies: 11.6% vs. 39.1% (p = 0.001) in ours and 10% vs. 25% (p = 0.003) in theirs. Furthermore, each 1 mm increase in glans size decreased complication odds in both of the studies: OR = 0.6 and 95% CI = 0.5-0.9 in ours and OR = 0.8 and 95% CI = 0.7-0.9 in Snodgrass et al.'s study. Interestingly, the same authors increased the glans' diameter from 12 mm to 16.5 mm via testosterone injection but did not achieve a reduction in complication rates [4]. In our multivariate analysis, the predictor value of glans size was independent of the child's age in accordance with previous studies [9].

Unlike our study, in two studies including proximal hypospadias cases but mostly distal hypospadias cases, age > four years and no coverage layer were the independent predictors of complications in another study by Eassa et al. [10]. Another study confirmed proximal hypospadias and double-layer closure (only urethroplasty layers) as independent predictors of complication while excluding age [11]. Since proximal hypospadias cases were excluded, we could not make a comparative analysis with the before-mentioned studies in terms of meatus location. However, it should be emphasized that distal hypospadias has a higher complication rate than mid-shaft hypospadias in our study. We hypothesize that distal glans surgery may cause smaller urethral caliber relative to mid-shaft hypospadias and higher complications consequently since the majority of our complications were glans dehiscence and meatal stenosis. In addition, we also could not show the superiority of double-layer urethral closure in contrast to the other two studies [10,11].

Only five cases (3.3%) of fistulas were observed in our study, which followed a meta-analysis of 7485 non-proximal cases with 4% (95% CI = 3.1-5.0%) estimated fistula incidence [8]. Only operation time (OR = 0.83, p = 0.03) continued its significance in the multivariate model as smaller glans diameter (mm) and lower operation time (min) were the only predictors of fistula in our univariate analysis. In a multicentric study of 591 cases with a fistula rate of 15.3%, binary logistic regression analysis indicated old age, not putting a cystostomy, and greater splint size as predictors of fistula [12]. Urethral plate, glandular grove, and glans shape were listed as fistula predictors in a study investigating 162 TIPU cases with a total fistula complication rate of 15.2% [1]. In contrast to the previous study, urethral plate/glans width proportion and proximal hypospadias were proposed as predictor factors in a study of 442 patients with a 10.8% fistula rate [13]. Furthermore, glans diameter was the only significant predictor in a study investigating pre-incision urethral width [14]. There is no consensus about fistula predictors, and with our few fistula complications, our shorter operation time prediction may be a statistical error and needs further investigation.

GDC, also known as meatal retraction, is a relatively common complication of TIPU surgery and an important reason for redo surgeries [15]. The complication rates are reported in a wide range of 3-22% in the literature, but these rates drop to 0-4% for distal hypospadias [15]. Our total GDC constituted 12.7% of the patients but only 2% of the patients had complete GDC (complete separation of glans wings), which required a redo operation and the remaining 10% had only partial glans dehiscence (up to 2 mm opening in the ventral lip of neourethra). Our complete GDC of 2% was in accordance with the 0-5% range reported by Snodgrass et al.'s summary table and 2.1% (95% CI = 1.3%-2.9%] of Wu et al.'s meta-analysis [8,15]. The remaining 10% of incomplete gland dehiscence may be comparable with meatal retraction, the pooled estimate of which was reported as 3.4% (95% CI = 0.1-6.6%) in the same meta-analysis. The possible reasons for our higher partial GDC were strict definition criteria and attention to complications, leaving the ventral lip of neourethral meatus 1 mm open to decrease meatal stenosis, and a relatively low number of studies included in the meta-analysis (only three case series). Young age and small glans (<14 mm) were significant predictors of GDC in our univariate analysis, but only small glans kept their significance (OR = 3.4, p = 0.03) in our multivariate model. In Snodgrass et al.'s model, proximal hypospadias and redo surgery were demonstrated as significant predictors. While they rejected the role of suture type and testosterone-supplemented glans augmentation for GDC prevention, they reported that extensive glans wing mobilization and freeing glans wings from corpora up to 4 mm distally reduces GDC complications.

Although they found proximal hypospadias as the predictor, the GDC rates were paradoxically 2% and 4% for mid-shaft and distal hypospadias cases relatively. Similarly, all of our GDC cases were glans-coronal located (26% vs. 0%, p = 0.001) but it was not significant in multivariate analysis, probably due to the lack of any cases in the mid-shaft group.

The rate of meatus stenosis was 8.6% in our study but a variety of rates were reported in general literature such as 0.7%, 5%, and 15.4% [1,16,17]. Glans diameter threshold of <14 mm was the only significant predictor of stenosis in our univariate and multivariate analysis. All meatus stenosis complications were detected in the glans-coronal group (17.8% vs. 0%, p = 0.001), and the significance did not persist in our univariate/multivariate analysis, again probably due to the lack of any case in the mid-shaft group. Deep plate incision, limited plate tubularization (approximate ending in mid-glans), and non-sutured glans closure over neo-urethra were suggested by Snodgrass et al. for stenosis prevention [18]. An alternative proposed solution was the dorsal inlay tubularized incised plate incision grafting to achieve adequate meatus caliber [19]. Güler et al. listed the stenosis predictors as narrow urethral plate, shallow glandular groove, and small glans in their study. They argued that 3 mm deep meatus was not achievable in patients with flat urethral grooves, and distal urethral incision resulted in higher meatal stenosis [1]. Snodgrass et al. challenged the former argument and rejected the urethral groove type as a predictor of stenosis [4]. Our high stenosis rate may be explained by the early calibration testing of meatus (21 days), the high percentage of patients with small glans diameter (42.6%), and possible accompanying edema. These are also supported by the fact that all of the stenosis patients had a normal calibration performed two months after the initial calibration.

This study, being a retrospective one, has some inherited limitations. It was a single-surgeon study that allowed the comparison of two groups and analyzing predicting factors; however, more extensive, database-based studies in multiple centers to better advocate the results are recommended. Per its nature, the randomization of the patients could not be succeeded. The lack of cases in some of the complication arms may have resulted in type 2 statistical error. The timing of meatus calibration (21 days after surgery) might result in the overrepresentation of meatal stenosis due to concurrent meatal stenosis. A single surgeon and single center review are limited to this study. A multi-center study would be of great value in determining the validity of the findings from this study.

## Conclusions

TIPU technique continues to be a mainstream surgery for distal hypospadias with notable success and acceptable complication rates. Small glans (<14 mm) and meatal position are the only significant predictors of the overall complication rate. These two factors keep their significance in the subgroup analysis of major complications, such as glans dehiscence and meatal stenosis. Preoperative assessment of the glans' width and meatal position is advised to predict complication rates. Implementing surgical improvements suggested by the pioneers in this field should not be forgotten even though anatomical factors are important for patient selection and decision-making. Prospective and randomized controlled studies on this subject may provide a better explanation of the subject.
